# Small Bowel Obstruction as a Complication of Uterine Fibroids: A Case Report

**DOI:** 10.7759/cureus.36902

**Published:** 2023-03-30

**Authors:** Rihab Farooq, Ammara S Sahibole, Nafeesathu Misiriyyah, Huda Ahmed, Haroutyoun Margossian

**Affiliations:** 1 Medicine, Dubai Academic Health Corporation, Dubai, ARE; 2 Obstetrics and Gynaecology, Dubai Academic Health Corporation, Dubai, ARE; 3 Urogynecology, Dubai Academic Health Corporation, Dubai, ARE

**Keywords:** obstetrics-gynecology, myomectomy, femoro-iliac venous thrombosis, pedunculated subserosal fibroid, small-bowel obstruction, uterine fibroid

## Abstract

Uterine fibroids are a common, benign gynecological smooth muscle neoplasm highly prevalent in women of reproductive age which can lead to multiple complications including small bowel obstruction.

We report a case of a 31-year-old female, primigravida at 13 weeks gestation, a known case of uterine subserosal fibroids who presented to the emergency with complaints of dark red vaginal bleeding and cramping abdominal pain. On examination, her abdomen was 38 weeks in size. An abdominal ultrasound showed intrauterine retained products of conception measuring 5x5 cm. She was admitted as a case of incomplete miscarriage and underwent an evacuation of retained products of conception (ERPOC) immediately.

A post-procedure computed tomography (CT) scan done showed the presence of multiple large uterine fibroids. The patient continued to worsen clinically with complaints of abdominal pain and diarrhea. Further laboratory tests revealed a continuous rise in inflammatory markers with positive stool clostridium toxins. She was hence shifted to the intensive care unit (ICU) as a case of sepsis. In the following days, she developed signs and symptoms of small bowel obstruction, and the diagnosis was supported by abdominal X-rays. Despite starting her on conservative management for the same, she deteriorated clinically, and a repeat CT abdomen showed new signs of small bowel obstruction. The gynecology team performed an exploratory laparotomy during which a myomectomy was done. The patient recovered well post-operatively and was discharged in a stable condition.

In view of the presented case, small bowel obstruction should be considered as a complication of uterine fibroids in females with a history of large leiomyomas despite it being quite rare as it can cause considerable morbidity and mortality.

## Introduction

Uterine fibroids are common, benign smooth muscle tumors that typically present with abnormal uterine bleeding, pelvic fullness, and subfertility or infertility [1]. However, these symptoms are seen in only 25% of patients. Increased risk is associated with older age, nulliparity, and obesity among others [2]. Bowel obstruction as a complication of uterine fibroids is rarely found in documented literature, making it quite a rare incidence. Adhesions are the most common risk factor for small bowel obstruction with masses and neoplasms accounting for a significantly lesser number of cases [3]. Transvaginal ultrasound imaging is the best diagnostic modality for fibroids while a computed tomography (CT) scan shows an adequate picture of the cause in most bowel obstruction scenarios [3,4]. Management of fibroids typically involves hormone modulation in some form and possible surgical interventions [4]. Meanwhile, the mainstay of management for bowel obstruction is relieving the obstruction, either with conservative measures like nasogastric decompression or with surgical options like adhesiolysis, and bowel resection with or without myomectomy if caused by uterine fibroids as in this case [3].

## Case presentation

A 31-year-old female, a known case of uterine subserosal fibroids with a term-sized abdomen, presented to the emergency at 13 weeks gestation with complaints of dark red vaginal bleeding, cramping abdominal pain, and the reported passage of a well-formed fetus followed by pieces of placenta one hour prior. She was a primigravida with spontaneous conception and no other significant medical or surgical history.

On arrival, she was vitally stable except for tachycardia of 117 beats per minute. On physical examination, she appeared pale. Abdominal examination revealed a hard mass of irregular shape occupying the abdomen, with a symphysio-fundal height of approximately 36 cm. On digital vaginal examination, the cervical os was open and fresh bleeding was seen with products of conception felt in the os. Blood tests done in the emergency are summarized in Table 1 which showed low hemoglobin and high white blood cell (WBC) count. In addition to that, the activated partial thromboplastin time (APTT) and fibrinogen level were elevated. On bedside ultrasound scan (USS), intrauterine retained products of conception measuring 5x5 cm were confirmed.

**Table 1:** Laboratory tests ordered in the emergency department on presentation WBC: white blood cells; RBC: red blood cells; MCV: mean corpuscular volume; MCH: mean corpuscular hemoglobin; MCHC: mean corpuscular hemoglobin concentration; RDW: red cell distribution width; INR: international normalised ratio; APTT: activated partial thromboplastin time.

Laboratory test	Laboratory value	Normal reference range
WBC count	14.2 x 10^3^/uL	3.6 - 11.0 x 10^3^/uL
RBC count	3.44 x 10^6^/uL	3.80 - 4.80 x 10^6^/uL
Hemoglobin, blood	8.6 g/dL	12.0 - 15.0 g/dL
Hematocrit	26.7%	36.0 - 46.0%
MCV	77.5 fL	77.0 - 95.0 fL
MCH	25.1 pg	27.0 - 32.0 pg
MCHC	32.3 g/dL	31.5 - 34.5 g/dL
RDW	14.9%	11.5 - 14.0%
Platelets count	467 x 10^3^/uL	150 - 410 x 10^3^/uL
Fibrinogen	1015 mg/dL	190 - 430 mg/dL
Prothrombin time	14.3 seconds	11.5 - 14.5 seconds
INR	1.11	0.80 - 1.20
APTT	47.7 seconds	28.6 - 38.2 seconds

She was diagnosed as a case of incomplete miscarriage and underwent an urgent evacuation of retained products of conception (ERPOC). The procedure was successful and completed with per rectal misoprostol 800 mcg insertion to control post-procedure bleeding. Post-operatively, she was vitally stable, she received two units of packed red blood cells (RBCs) to improve her pre-procedural hemoglobin and was started on dual antibiotics, doxycycline and metronidazole.

**Figure 1:**
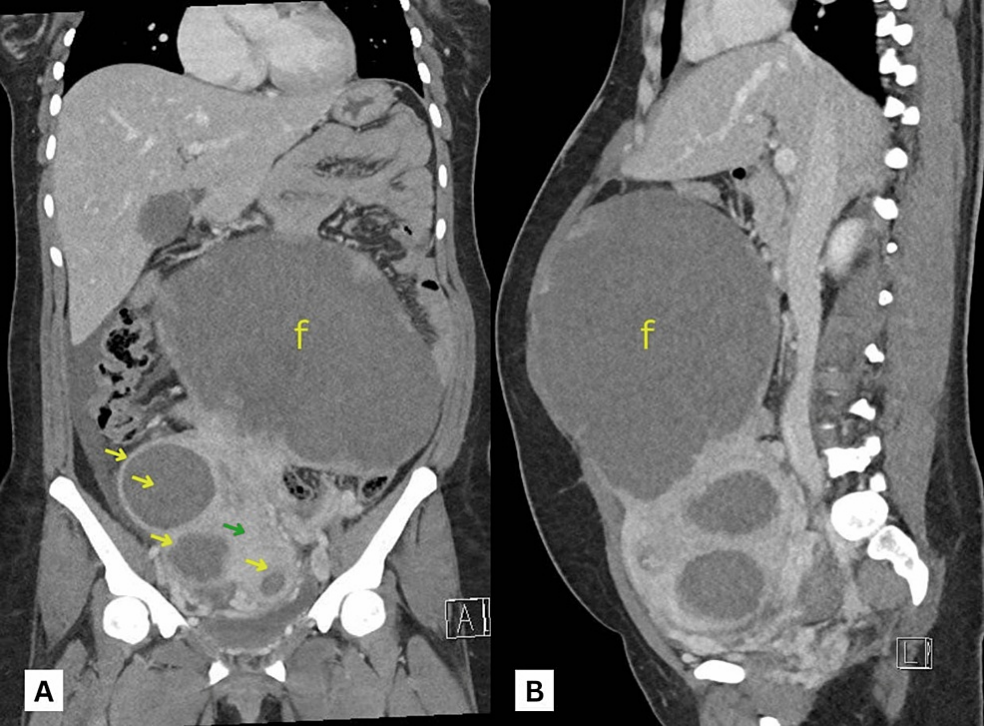
Abdominal CT scan images: (A) coronal view and (B) sagittal view, which show multiple uterine fibroids (yellow arrows) with single large subserosal leiomyoma (f) pushing the bowels superior and laterally Green arrow: uterus

Despite an initial improvement in her symptoms, on post-operative day 1, she developed worsening abdominal pain and distension. Biochemistry showed elevated inflammatory markers namely white blood cell count (WBC), C-reactive protein (CRP) and procalcitonin. Hence, a computed tomography (CT) scan was done due to suspicion of uterine perforation which showed multiple large uterine degenerated leiomyomas pushing the small and large bowels superiorly and laterally (Figure 1A, 1B). An attenuated left ovarian vein with no definitive thrombosis, compression of the proximal left common iliac vein due to the large fibroid uterus, and a left femoro-iliac vein thrombosis could also be appreciated. Moderate amounts of intra-abdominal fluid and no intra-abdominal free air were seen.

Based on the CT findings of the left femoro-iliac vein thrombosis, she was started on a therapeutic dose of low-molecular-weight heparin, and vascular surgery was consulted who advised to continue anticoagulation with no other intervention required.

In the following few days, inflammatory markers continued to increase, and the patient became tachypnoeic while complaining of worsening abdominal pain, diarrhea, and bilious vomiting. Laboratory investigations done to identify the source of infection revealed a positive stool clostridium toxins A and B test. A diagnosis of sepsis was established, and the patient was shifted to the intensive care unit (ICU) for optimal management.

**Figure 2:**
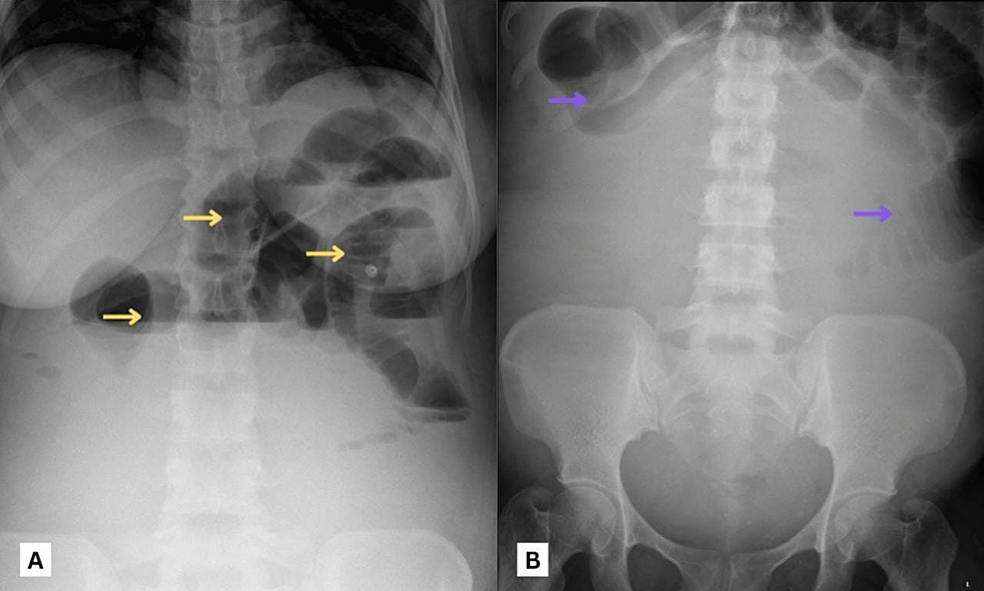
X-ray images of the abdomen showing signs of bowel obstruction: (A) Erect view with multiple air-fluid levels (yellow arrows) without pneumoperitoneum. (B) Supine view with dilated small bowel loops (purple arrows)

**Figure 3:**
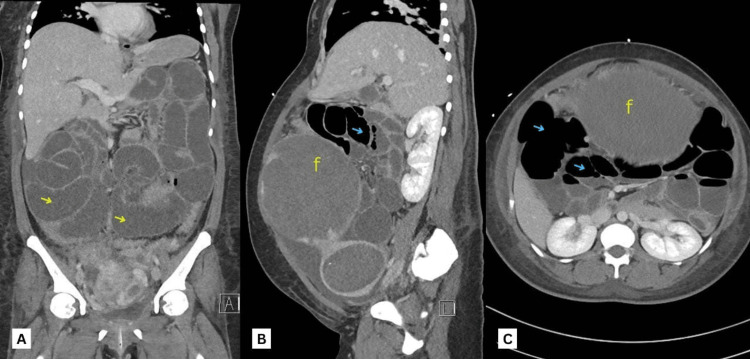
Abdominal CT scan images showing signs of bowel obstruction: (A) Coronal view with dilated small bowel loops (yellow arrows). (B) Sagittal view and (C) transverse view with multiple air-fluid levels (blue arrows) f: fibroid

Antibiotic therapy was upgraded to metronidazole and meropenem. The vascular surgeon advised the placement of an inferior vena cava (IVC) filter if the patient was to be operated on. The patient failed to pass flatus for the next 24 hours. Physical examination revealed a distended abdomen with generalized tenderness to palpation and increased bowel sounds on auscultation. In view of her symptoms and physical exam findings, an abdominal X-ray was done that revealed the presence of multiple air-fluid levels with dilated bowel loops suggestive of small bowel obstruction (Figure 2A, 2B). The general surgeon was consulted due to the suspicion of a small bowel obstruction and was advised to keep the patient nil per os (NPO) with intravenous (IV) fluid infusion and a nasogastric tube (NGT) insertion for decompression. The following day, she further developed an inability to pass stool and continued to worsen clinically. An urgent CT abdomen showed new signs of small bowel obstruction (Figure 3A-3C) and dissolution of the large venous thrombosis.

**Figure 4:**
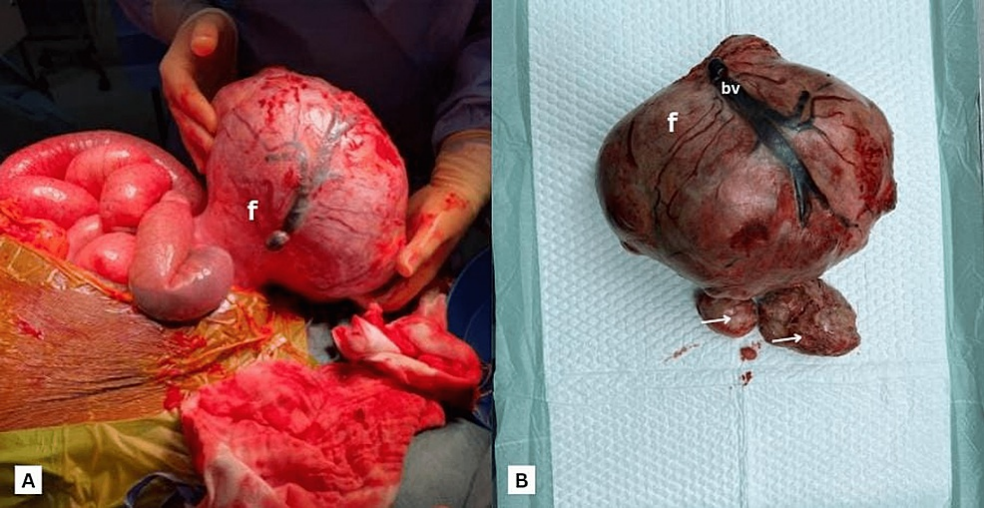
Intra-operative images showing (A) large subserosal leiomyoma before resection, and (B) after resection, with prominent blood vessel on the surface (bv), smaller fibroids (white arrow) f: fibroid

Despite the advice of the gynecology team to perform an exploratory laparotomy and a total abdominal hysterectomy, the patient and her partner chose the fertility-preserving option of abdominal myomectomy instead. A large pedunculated fibroid weighing 3.5 kg with a large vessel on the surface and attached to the anterior left lateral uterine fundus was intra-operatively removed along with two other fibroids measuring 5 cm each (Figure 4A, 4B). The omentum and small bowel loops were found densely adherent to the fibroid, hence confirming the diagnosis of small bowel obstruction. A right ovarian cystectomy was done due to the intraoperative finding of an ipsilateral ovarian endometrioma. No endometriotic lesions were seen on the bowel surface, adequately ruling it out as a cause of the bowel obstruction. Bowel run was done during the procedure which revealed normal bowels and did not require resection or anastomosis. IVC filter was not placed as the CT scan showed dissolution of the left femoro-iliac venous thrombus subsequent to adequate anticoagulation. She later received two units of fresh frozen plasma (FFP) and one unit of whole blood. Post-operatively, she recovered well with no early surgical or other complications and was discharged one week later with continued anticoagulation and a follow-up appointment in the gynecology clinic.

## Discussion

Uterine fibroids are a gynecological benign smooth muscle neoplasm with an incidence on the rise, affecting around 25% of women in their reproductive age while causing symptoms in around 25% of the patients [1]. The patient we report in this case was asymptomatic as is more common with this condition. These symptoms include abnormal uterine bleeding (AUB), pelvic fullness, or affecting fertility [1]. One extremely rare presentation is bowel obstruction with relatively few cases documented in the literature. One of these documented cases involved a patient with a known history of fibroids and no surgical history presenting with abdominal pain [5]. Another case reported small bowel obstruction occurring after a cholecystectomy due to a massively enlarged fibroid uterus [6].

Increased risk is identified in African descent, a positive family history, women older than 40 years of age, nulliparity, and obesity [2]. While adhesions remain the top most common risk factor for bowel obstruction, masses and neoplasms account for 5% of cases, with fibroids falling under the same category [3].

The clinical presentation is usually related to the location, type, and size of the mass. While history and physical exam guide adequately to the diagnosis, a significant amount of asymptomatic cases have resulted in transvaginal ultrasound imaging being considered the first and most sensitive diagnostic modality for this condition [4]. With respect to bowel obstruction, computed tomography (CT) scan is superior in terms of its advantage of guiding to the cause of the obstruction like the fibroids in our case here [3].

Management of uterine fibroids is multifactorial, depending on size, symptoms, type, and most importantly, desire for fertility. Conservative therapy revolves around hormonal manipulation in the form of Gonadotropin-releasing hormone (GnRH) agonists and GnRH antagonists, oral contraceptives, and progesterone receptor modulators alongside non-steroidal anti-inflammatory drugs (NSAIDs) and tranexamic acid. Surgical therapy encompasses myomectomy, uterine artery embolization, and lastly, hysterectomy [4]. In the setting of a uterine fibroid causing bowel obstruction, standard approaches like nasogastric decompression, keeping the patient nil per os (NPO), hydration, and analgesia are implemented. If the patient fails to improve with conservative measures, surgical treatment is indicated in the form of adhesiolysis or myomectomy as was done in our case, or hysterectomy depending on the patient's desire for fertility [3]. Relieving the cause of obstruction becomes the cornerstone of management in this scenario. Resection of gangrenous and necrotic bowel, if any, is also performed depending on intraoperative findings.

## Conclusions

Despite bowel obstruction being a rare complication of uterine fibroids, it can be fatal if not promptly diagnosed and managed early, causing considerable morbidity and mortality. Diagnosis of this complication becomes even more challenging if the concerned fibroid(s) is large enough to occupy the abdominal cavity and displace the bowel. When dealing with such large fibroids, large vein thrombosis also needs to be considered and evaluated for as it may increase the morbidity of any surgical intervention. This condition needs to be addressed and managed adequately before any surgery is performed. Small bowel obstruction as a complication of uterine fibroids, therefore, requires better reporting in literature. More research and extensive studies are needed to have a better understanding of the nature of this condition.
